# P10-12 DIPPAO: digital-based intervention promoting physical activity among obese people

**DOI:** 10.1093/eurpub/ckac095.151

**Published:** 2022-08-29

**Authors:** Alexandre Mazeas, Aïna Chalabaev, Marine Blond, Martine Duclos

**Affiliations:** 1 SENS Laboratory, University Grenoble Alpes, Grenoble, France; 2 INRA, UMR 1019, Clermont-Ferrand, France; 3 Kiplin, Nantes, France; 4 Department of Sport Medicine and Functional Explorations, Clermont-Ferrand University Hospital, G. Montpied Hospital, Clermont-Ferrand, France

**Keywords:** e-health, gamification, obesity, stereotypes, behavior change

## Abstract

Despite the accumulation of scientific evidence supporting the benefits of physical activity, the general population remains insufficiently active and this situation is even worsened for some specific populations such as obese people (Barker et al., 2019). It is therefore becoming urgent to develop interventions that can effectively change individuals' behavior. In this context, “e-health” interventions and gamification appear to be a particularly promising avenue to improve physical activity and reduce attrition rates of current programs.

This study protocol aims to present a randomized, two-arm intervention design that will examine the efficacy of a digital intervention based on play and teamwork for an obese sample. Fifty patients will be recruited at the hospital of Clermont-Ferrand, France and randomized into two groups: the experimental group will receive digital supervised sessions of PA in videoconference and a gamification of PA through a mobile app and will be compared to an active control group representing the traditional care program (supervised physical activity three times per week during three months). Informed by the tenets of the social identity approach and built around behavior change techniques, implemented via the principles of gamification, this three-month intervention has the objective to improve physical activity adherence. Objective and self-reported PA, quality of live, physical condition and psychological variables like motivation or perceived discrimination will be assessed before, at the end of the intervention and then at the issue of a 6-month follow up in order to evaluate the long-term effect of the intervention. We make the assumption that the intervention will be efficient by the development of an intrinsic motivation through the process of gamification on the one hand. On the other hand, through the in-group collaboration with other people who share the same stigmatized criteria that will help participants to overcome weight stigmas, acting generally as physical activity barriers.

This poster presentation will introduce the conception of the intervention, the experimental design and proposed evaluation of the trial. If the effectiveness of this intervention is confirmed, its implementation in the health circuit could reduce the costs and constraints associated to face-to-face support.

